# Novel equi merozoite antigen (*ema-1*) gene heterogeneity in a geographically isolated *Theileria equi* population in Croatia

**DOI:** 10.1186/s13071-022-05484-4

**Published:** 2022-10-31

**Authors:** Robert Coultous, Jelena Gotić, Martine McCann, David Sutton, Relja Beck, Brian Shiels

**Affiliations:** 1 Institute of Biodiversity Animal Health and Comparative Medicine, University of Glasgow, Glasgow, UK; 2 Internal Diseases Clinic, Faculty of Veterinary Medicine, University of Zagreb, Zagreb, Croatia; 3 School of Veterinary Medicine, University of Glasgow, Glasgow, UK; 4 Laboratory for Parasitology, Croatian Veterinary Institute, Zagreb, Croatia

**Keywords:** Equine, Piroplasmosis, Croatia, *Theileria equi*, *Ema-1*, Equi merozoite antigen

## Abstract

**Background:**

The apicomplexan haemoparasite *Theileria equi*, a causative agent of equine piroplasmosis, is an established pathogen of significant welfare and economic concern within the Croatian equine population. A previous large surveillance study of *T. equi* has identified two distinct parasite populations, one in the north and one in the south, geographically separated by the Dinaric Alps, which traverse the country. This study aimed to further investigate the genetic diversity within these two populations, focussing on allelic variability of the equi merozoite antigen gene, *ema-1*.

**Methods:**

Following nested PCR of DNA isolates, the generated *ema-1* amplicons were subsequently sequenced and compared by phylogenetic analysis to available sequences representing previously described *ema-1* genotypes (groups A–C).

**Results:**

Isolates from the southern *T. equi* population clustered with the existing *ema-1* groups A and B. Strikingly, isolates from the northern population clustered into two novel *ema-1* genotypes, named groups D and E.

**Conclusions:**

This detection of hitherto unreported genotypes suggests that historic geographical isolation has led to a degree of divergent evolution in this northern *T. equi* population. Additionally, current global regulatory testing of equine piroplasmosis relies heavily on EMA-1 based immunodiagnostics, and the discovery of unique *ema-1* genotypes may question the efficacy of current diagnostics in international equine movement, with ramifications for the global equine community.

**Graphical Abstract:**

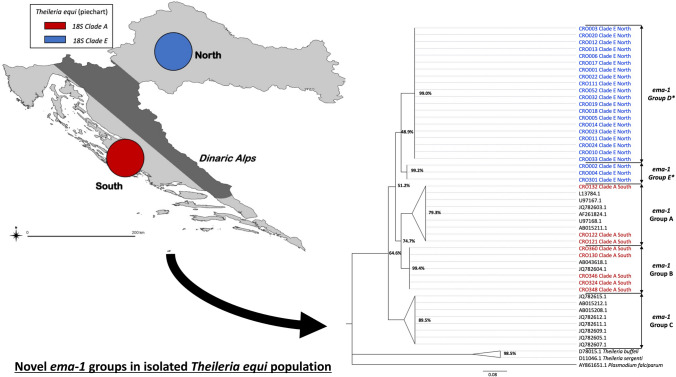

## Background

*Theileria equi* has long been established as a pathogen of significant welfare and economic concern within the Croatian equine population [1]. The pathogen is one of the tick-borne apicomplexan parasites responsible for equine piroplasmosis, a disease with ubiquitous global presence which presents in three main clinical forms: acute or sub-acute disease with severe anaemia, pyrexia and dehydration, with death occurring in severe cases; chronic disease with animals displaying fluctuating malaise, weight loss or reduced performance [2]. Importantly, infected animals become carriers for life [3], acting as reservoirs for further infection and demonstrating disease recrudescence in times of illness or stress [4, 5].

Current research has established that *Theileria equi* demonstrates an unusually high degree of genetic heterogeneity at the 18S rRNA gene compared to other *Theileria* species [6], and recently a separate species, *Theileria haneyi*, has been identified within the *Theileria equi* umbrella [7]. A five-clade (A–E) molecular genotyping system has been established based on this 18S rRNA gene locus [8, 9] and has been used extensively in numerous *T. equi* surveillance studies in Europe [10, 11], Africa [12], the Americas [13] and the Middle East [14]. Other *Theileria* species, such as *Theileria annulata* and *Theileria parva*, show a high degree of genetic diversity in geographical areas with long-standing parasitic populations [15, 16]. Previous studies have shown this to also be true for *T. equi*, with historically endemic areas demonstrating not only multiple parasite genotype clades within the equine population [12, 17], but also a multiplicity of infection with individuals [17], whereas areas of more recent parasite introduction can show the presence of only a single clade [10].

**Fig. 1:**
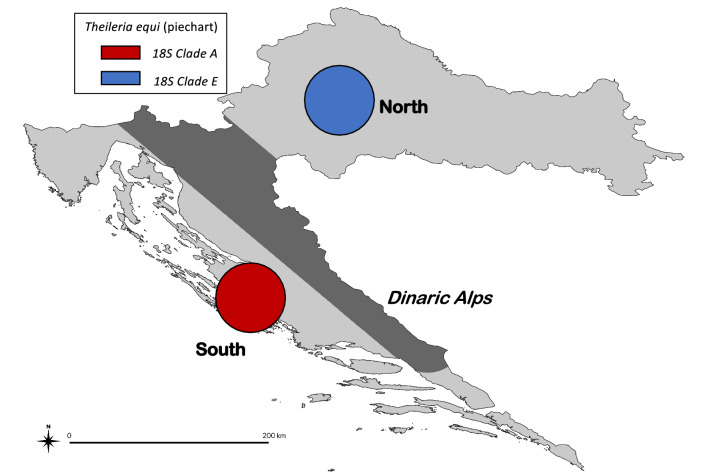
The geographical distribution of *Theileria equi* 18S rRNA clade types within samples collected in northern and southern Croatia. The mountainous area dividing the two regions is marked in grey

As part of a larger surveillance study of piroplasmosis within the Croatian equine population [18], *T. equi* DNA was identified by PCR in 48 blood samples from equines across Croatia using previously described 18S rRNA gene specific primers [19]. Sequencing of generated amplicons showed the presence of just two *T. equi* 18S rRNA gene clades, A and E. When the geographical residence of these sampled equines was mapped, a distinct geographical separation of the clades was noted with clade E samples found exclusively in the north and clade A samples restricted to the south, with both populations separated by the Dinaric Alps which traverse Croatia (Fig. 1). This geographic separation is extremely unusual given the heterogenous mixing of clades seen in other historically endemic countries [12, 17]. Given the importance of the disease to the Croatian equine industry, and the endemic presence of the parasite within the country, this finding warranted further, more detailed investigation of the genetic diversity within these two *T. equi* populations.

The equi merozoite antigen-1 (*ema-1*) is a surface exposed protein, expressed during the erythrocytic stage of *T. equi* infection and utilised extensively as a target for commercial ELISA detection [20]. Although initially thought to be well conserved within the species [21, 22], further research has identified variability with three defined *ema-1* groups defined [9, 23]. The heterogeneity at this locus makes this an ideal gene for assessing further genetic diversity within *T. equi* clade populations.

This report describes the methods and findings following investigation of *ema-1* diversity within the two geographically separated Croatian *T. equi* populations.

## Methods

DNA was extracted using a commercial extraction kit (DNeasy blood & tissue kit, Qiagen, Germany) from equine blood samples previously collected between 2012 and 2014 as part of a larger national equine haemoparasite surveillance study in Croatia. A total of 32 samples were available from the original 48 samples previously identified as containing *T. equi* DNA following screening with a *Theileria*/*Babesia* spp. 18S rRNA gene targeting PCR protocol [19]. Sequencing of the generated amplicons allowed clade genotyping based on this locus.

These 32 DNA samples were then further screened using novel primers targeting a hyper variable region of the *ema-1* gene in a nested PCR protocol (Table 1). Reaction conditions for the first round of the PCR were a final volume of 20 μl with 5 × GoTaq Colorless Reaction Buffer (Promega, USA) (final concentrations of 1.5 mM MgCl2), Deoxynucleotide (dNTP) Solution Mix (New England Biolabs, USA) (final concentrations of 0.2 mM dATP, 0.2 mM dCTP, 0.2 mM dGTP and 0.2 mM dTTP), 0.5 μM of each outer primer (RC_EMA_F1 and RC_EMA_R1), 0.025 units/μl DNA polymerase (GoTaq G2, Promega, USA) and 2 μl of template genomic DNA solution.

**Table 1:** Details of the *ema-1* specific primers used in this study

Primer name	Primer pair	Sequence	Expected amplicon size (base pairs)
RC_EMA_F1	Outer	5′–GACTTGAAGGAACADACCCC–3′	
RC_EMA_R1	Outer	5′–AGGTCGAGRACAACTTCGTT–3′	298
RC_EMA_F2	Inner	5′–GGCACATCAAGGACAACAAGCC–3′	
RC_EMA_R2	Inner	5′–CGCTTGCCTGGAGCCTTGAA–3′	197

The cycling conditions for the primary reaction were an initial denaturation of 94 °C for 5 min, then 30 cycles of denaturation at 94 °C for 30 s, annealing at 50 °C for 30 s, and extension at 74 °C for 60 s, with a final extension at 72 °C for 5 min. Reactions for the secondary PCR were performed using identical reagent concentrations and cycling conditions except using the inner primer pair (RC_EMA_F2 and RC_EMA_R2). A 1:10 dilution of the primary reaction product was used as the DNA template in the secondary PCR reaction. The final product was visualised using gel electrophoresis with a 1% agarose gel. The PCR product was purified (QIAquick PCR purification kit, Qiagen, Germany) and submitted for Sanger sequencing (Eurofins Genomics, Germany). Amplicons were sequenced in both directions with a consensus sequence generated. Known positive and negative samples from a previous study were used as controls [10].

Species identification of sequences obtained in this study was achieved using the basic local alignment search tool (BLAST) and comparison with sequences deposited in the non-redundant National Center for Biotechnology Information (NCBI) GenBank database (https://blastncbi.nlm.nih.gov/). The MUSCLE function [24], within the AliView alignment viewer and editor [25], was used to compare the study sequences with those previously determined and deposited in the NCBI GenBank database. Nucleotide diversity statistics were generated using DnaSP [26].

A maximum likelihood phylogenetic tree was constructed using MEGA11 software [27] to compare the genetic diversity of the *ema-1* gene sequences generated from the study samples with previously defined *ema-1* groups [9, 23], based on 1000 replications. Piroplasm surface protein gene sequences of *Theileria buffeli* (D78015) *and Theileria sergenti* (D11046) and a *Plasmodium falciparum* surface antigen (AY861651) were included in the trees as outgroups [23]. All sequences generated in this report were submitted to the NCBI GenBank database (https//www.ncbi.nlm.nih.gov/genbank/) accession numbers ON502853—ON502883 inclusve.

## Results

Amplicons were generated and successfully sequenced from 31 of the 32 available equine DNA samples, and BLAST analysis demonstrated all amplicons had an identity of between 92 and 100% with existing *T. equi ema-1* sequences in the NCBI GenBank database.

**Fig. 2:**
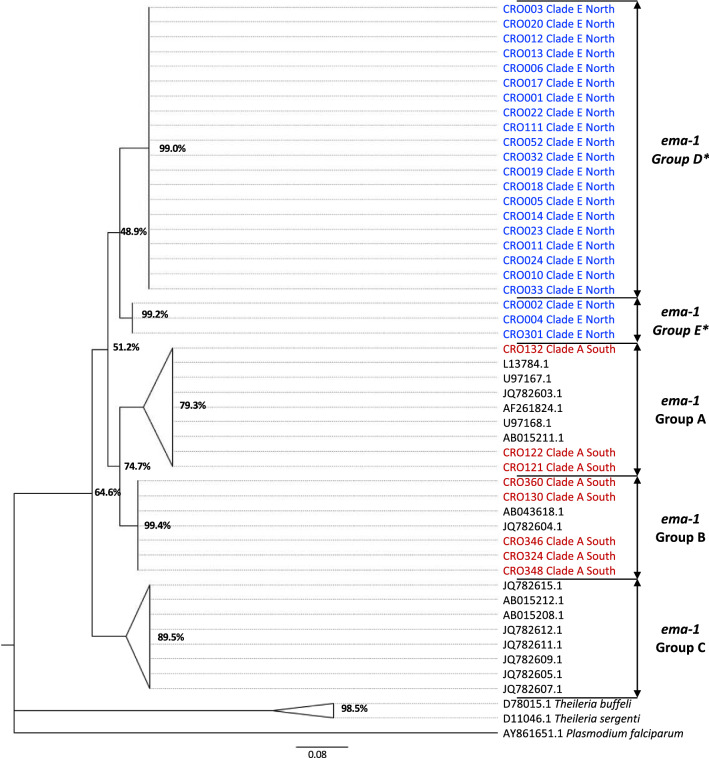
A maximum-likelihood tree inferring the evolutionary relationship of the *ema-1* sequences from the *Theileria equi* samples identified in this survey. Included are all detected *T. equi* sequences from northern Croatia (blue) and southern Croatia (red). Other representative sequences of *T. equi*, *Theileria buffeli*, *Theileria sergenti* and *Plasmodium falciparum* (to which the tree is rooted) are included with GenBank accession numbers. Bootstrap values are shown as a percentage, based on 1000 replications. The previously described *ema-1* groups, **A**–**C**, and the novel *ema-1* groups, **D**, **E**, have been annotated, and external branches have been collapsed to the clade level

The maximum-likelihood phylogenetic tree constructed to compare the Croatia *ema-1* sequences with the previously defined *ema-1* groups is shown in Fig. 2.

All samples from southern Croatia, previously identified as belonging to the 18S rRNA gene clade A, demonstrated *ema-1* homology to the previously described *ema-1* genotype groups A and B. All samples from northern Croatia, previously identified as belonging to the 18S rRNA gene clade E, were assembled distinct from the previously described *ema-1* groups and clustered in to two novel genotype groups, tentatively named *ema-1* groups D and E.

To investigate the variation in diversity between the samples from the different regions at the sequence level, within-region expected heterozygosity (He) was calculated using the allelic sequences derived from the northern Croatian, southern Croatian and *ema-1* reference samples (Table 2). A relatively lower heterozygosity (He 0.24) was evident in the northern sample set compared to those in the south (He 0.64) and the reference samples (He 0.93). In addition, the nucleotide diversity (π) and average number of differences between samples (k) were calculated for each group (Table 2). This analysis further highlighted the reduced *ema-1* diversity among the northern Croatian samples, which contrasted to that of the southern Croatian samples and those from other countries which showed substantial variation at the nucleotide level.

**Table 2:** Analysis of genetic diversity at the *ema-1* locus present within samples from Croatia and other geographical origins

Sequence group	No. sequences	No. haplotypes	Heterozygosity (He)	Nucleotide diversity (pi)	Average number of differences between samples (k)
Croatia North	23	2	0.237	0.0142	2.372
Croatia South	8	4	0.643	0.0289	4.821
*ema-1* reference	16	10	0.925	0.0929	10.317

## Discussion

The geographic distribution of *T. equi* phylogeny in Croatia is unique compared to that of other endemic countries [11], with a northern population clearly derived from the 18S rRNA clade E and a southern population derived from 18S rRNA clade A, separated by the robust geographic boundary of the Dinaric Alps.

The findings of the *ema-1* phylogeny in this report show a homology between the southern *T. equi* population and previously defined *ema-1* groups from global samples. This suggests the southern population may be a result of introduction from different geographic areas, most likely from historical international movement of horses into Croatia.

In contrast, the northern *T. equi* samples revealed the identification of two novel *ema-1* genotypes. The detection of multiple novel gene groups implies that geographic isolation of this northern population has been maintained for a substantial chronological period. The northern region has a continental climate and geography, where the tick species of *Dermacentor reticulatus, Dermacentor marginatus* and *Rhipicephalus sanguineus* predominate, contrasting with the southern region which possesses a coastal climate and geography where *Hyalomma marginatum*, *Rhipicephalus bursa* and *Rhipicephalus turanicus* populations exist as [28]. The unique selection pressures of the northern region, likely a combination of the environment, presence of local tick species and equine host availability, has led to a degree of divergent evolution and development of unique genotypes within this isolated population.

As well as providing a distinctive example of divergent evolution in a *T. equi* population, these results further expand on the complexity of the species’ phylogeny being depicted in recent literature [9]. The relative homogeneity of the northern *T. equi* population and unique *ema-1* genotypes may even be suggestive of a separate subspecies or species, such as the recently described *T. haneyi*, which clusters with the *T. equi* 18S rRNA clade C but has been demonstrated to lack the *ema-1* gene completely [7]. However, this conclusion is beyond the scope of the results presented in the current report.

The results of this report also have relevance to the global equine community. Current regulatory testing of equine piroplasmos is heavily on EMA-1 based cELISA diagnostics [20], and despite reduced efficacy of commercial EMA-1 diagnostics having already been suggested within the previously known *ema-1* genotypes [23], this method is still recommended by the World Organisation for Animal Health (OIE) for use in international equine movement [29]. The presence of the additional and substantial *ema-1* heterogeneity described in this report further questions this existing dependence on EMA-1 as a target for immunodiagnostic screening, especially when the national biosecurity of countries without endemic equine piroplasmosis is at risk and introduction of the disease can lead to prolonged and costly eradication [30]. In light of this increasing evidence of diversity at this locus, newer diagnostics now additionally combine EMA-2 detection as part of immunodiagnostic screening methods [9, 31], and the international equine community would be wise to continually evaluate the robustness of current protocols and ideally seek more conserved, but specific targets for diagnostic testing.

The relatively small sample size, especially the available samples from southern regions, is a limitation of this study, and consequently the findings may not be representative of the country as a whole. In addition, the role of iatrogenic transmission events, which can be significant in the spread of infection [30], could not be assessed from the available data. However, given the infrequent occurrence of these events, and the wide-geographical area over which the isolates and novel genotypes have been detected, any iatrogenic transmission is unlikely to have had a significant impact on the reported findings.

## Conclusion

This study has identified two hitherto unreported *ema-1* genotypes suggesting that historic geographical isolation in Croatia has led to a degree of divergent evolution between the northern versus southern *T. equi* population. In addition to expanding current scientific understanding of *T. equi* species diversity, the discovery of unique *ema-1* genotypes adds to current equine community concerns over the efficacy of current diagnostics in international equine movement.

## Data Availability

The datasets generated and analysed during the current study are available in the NCBI GenBank repository (accession numbers ON502853—ON502883 openly accessible at https://www.ncbi.nlm.nih.gov/genbank/).

## Abbreviations

18S rRNA: 18S (Svedberg units) small subunit ribosomal ribonucleic acid
BLAST: Basic local alignment search tool
cELISA: Competitive enzyme-linked immunosorbent assay
EMA-1: Equi merozoite antigen one protein
*ema-1*: Equi merozoite antigen one gene
OIE: World Organisation for Animal Health
